# The Effect of Antibiotic Exposure and Specimen Volume on the Detection of Bacterial Pathogens in Children With Pneumonia

**DOI:** 10.1093/cid/cix101

**Published:** 2017-05-27

**Authors:** Amanda J. Driscoll, Maria Deloria Knoll, Laura L. Hammitt, Henry C. Baggett, W. Abdullah Brooks, Daniel R. Feikin, Karen L. Kotloff, Orin S. Levine, Shabir A. Madhi, Katherine L. O’Brien, J. Anthony G. Scott, Donald M. Thea, Stephen R. C. Howie, Peter V. Adrian, Dilruba Ahmed, Andrea N. DeLuca, Bernard E. Ebruke, Caroline Gitahi, Melissa M. Higdon, Anek Kaewpan, Angela Karani, Ruth A. Karron, Razib Mazumder, Jessica McLellan, David P. Moore, Lawrence Mwananyanda, Daniel E. Park, Christine Prosperi, Julia Rhodes, Md. Saifullah, Phil Seidenberg, Samba O. Sow, Boubou Tamboura, Scott L. Zeger, David R. Murdoch, for the PERCH Study Group, Katherine L. O’Brien, Orin S. Levine, Maria Deloria Knoll, Daniel R. Feikin, Andrea N. DeLuca, Amanda J. Driscoll, Nicholas Fancourt, Wei Fu, Laura L. Hammitt, Melissa M. Higdon, E. Wangeci Kagucia, Ruth A. Karron, Mengying Li, Daniel E. Park, Christine Prosperi, Zhenke Wu, Scott L. Zeger, Nora L. Watson, Jane Crawley, David R. Murdoch, W. Abdullah Brooks, Hubert P. Endtz, Khalequ Zaman, Doli Goswami, Lokman Hossain, Yasmin Jahan, Hasan Ashraf, Stephen R. C. Howie, Bernard E. Ebruke, Martin Antonio, Jessica McLellan, Eunice Machuka, Arifin Shamsul, Syed M. A. Zaman, Grant Mackenzie, J. Anthony G. Scott, Juliet O. Awori, Susan C. Morpeth, Alice Kamau, Sidi Kazungu, Micah Silaba Ominde, Karen L. Kotloff, Milagritos D. Tapia, Samba O. Sow, Mamadou Sylla, Boubou Tamboura, Uma Onwuchekwa, Nana Kourouma, Aliou Toure, Shabir A. Madhi, David P. Moore, Peter V. Adrian, Vicky L. Baillie, Locadiah Kuwanda, Azwifarwi Mudau, Michelle J. Groome, Nasreen Mahomed, Henry C. Baggett, Somsak Thamthitiwat, Susan A. Maloney, Charatdao Bunthi, Julia Rhodes, Pongpun Sawatwong, Pasakorn Akarasewi, Donald M. Thea, Lawrence Mwananyanda, James Chipeta, Phil Seidenberg, James Mwansa, Somwe wa Somwe, Geoffrey Kwenda, Trevor P. Anderson, Joanne Mitchell

**Affiliations:** 1 Department of International Health, International Vaccine Access Center, Johns Hopkins Bloomberg School of Public Health, Baltimore, Maryland;; 2 Kenya Medical Research Institute–Wellcome Trust Research Programme, Kilifi;; 3 Global Disease Detection Center, Thailand Ministry of Public Health–US Centers for Disease Control and Prevention Collaboration, Nonthaburi;; 4 Division of Global Health Protection, Center for Global Health, Centers for Disease Control and Prevention, Atlanta, Georgia;; 5 Department of International Health, Johns Hopkins Bloomberg School of Public Health, Baltimore, Maryland;; 6 International Centre for Diarrhoeal Disease Research, Bangladesh (icddr,b), Dhaka and Matlab;; 7 Division of Viral Diseases, National Center for Immunizations and Respiratory Diseases, Centers for Disease Control and Prevention, Atlanta, Georgia; 8 Division of Infectious Disease and Tropical Pediatrics, Department of Pediatrics, Center for Vaccine Development, Institute of Global Health, University of Maryland School of Medicine, Baltimore;; 9 Bill & Melinda Gates Foundation, Seattle, Washington;; 10 Medical Research Council, Respiratory and Meningeal Pathogens Research Unit, and; 11 Department of Science and Technology/National Research Foundation, Vaccine Preventable Diseases Unit, University of the Witwatersrand, Johannesburg, South Africa;; 12 Department of Infectious Disease Epidemiology, London School of Hygiene & Tropical Medicine, United Kingdom;; 13 Center for Global Health and Development, Boston University School of Public Health, Massachusetts;; 14 Medical Research Council Unit, Basse, The Gambia;; 15 Department of Paediatrics, University of Auckland, and; 16 Centre for International Health, University of Otago, Dunedin, New Zealand; Departments of; 17 Epidemiology and; 18 International Health, Center for Immunization Research, Johns Hopkins Bloomberg School of Public Health, Baltimore, Maryland;; 19 Cummings School of Medicine, University of Calgary, Alberta, Canada;; 20 Department of Paediatrics and Child Health, Chris Hani Baragwanath Academic Hospital and University of the Witwatersrand, South Africa;; 21 University Teaching Hospital, Lusaka, Zambia;; 22 Milken Institute School of Public Health, Department of Epidemiology and Biostatistics, George Washington University, Washington, District of Columbia; 23 Department of Emergency Medicine, University of New Mexico, Albuquerque;; 24 Centre pour le Développement des Vaccins (CVD-Mali), Bamako;; 25 Department of Biostatistics, Johns Hopkins Bloomberg School of Public Health, Baltimore, Maryland; and; 26 Department of Pathology, University of Otago, and; 27 Microbiology Unit, Canterbury Health Laboratories, Christchurch, New Zealand; 28 Johns Hopkins Bloomberg School of Public Health, Baltimore, Maryland; 29 Emmes Corporation, Rockville, Maryland; 30 Nuffield Department of Clinical Medicine, University of Oxford, United Kingdom; 31 University of Otago, Christchurch, New Zealand; 32 icddr,b, Dhaka and Matlab, Bangladesh; 33 Medical Research Council, Basse, The Gambia; 34 KEMRI-Wellcome Trust Research Programme, Kilifi, Kenya; 35 Division of Infectious Disease and Tropical Pediatrics, Department of Pediatrics, Center for Vaccine Development, Institute of Global Health, University of Maryland School of Medicine, Baltimore, Maryland and Centre pour le Développement des Vaccins (CVD-Mali), Bamako, Mali; 36 Respiratory and Meningeal Pathogens Research Unit, University of the Witwatersrand, Johannesburg, South Africa; 37 Thailand Ministry of Public Health–US CDC Collaboration, Nonthaburi, Thailand; 38 Boston University School of Public Health, Boston, Massachusetts and University Teaching Hospital, Lusaka, Zambia; 39 Canterbury Health Laboratory, Christchurch, New Zealand

**Keywords:** pneumonia, blood culture, antibiotic exposure, children.

## Abstract

**Background.:**

Antibiotic exposure and specimen volume are known to affect pathogen detection by culture. Here we assess their effects on bacterial pathogen detection by both culture and polymerase chain reaction (PCR) in children.

**Methods.:**

PERCH (Pneumonia Etiology Research for Child Health) is a case-control study of pneumonia in children aged 1–59 months investigating pathogens in blood, nasopharyngeal/oropharyngeal (NP/OP) swabs, and induced sputum by culture and PCR. Antibiotic exposure was ascertained by serum bioassay, and for cases, by a record of antibiotic treatment prior to specimen collection. Inoculated blood culture bottles were weighed to estimate volume.

**Results.:**

Antibiotic exposure ranged by specimen type from 43.5% to 81.7% in 4223 cases and was detected in 2.3% of 4863 controls. Antibiotics were associated with a 45% reduction in blood culture yield and approximately 20% reduction in yield from induced sputum culture. Reduction in yield of *Streptococcus pneumoniae* from NP culture was approximately 30% in cases and approximately 32% in controls. Several bacteria had significant but marginal reductions (by 5%–7%) in detection by PCR in NP/OP swabs from both cases and controls, with the exception of *S. pneumoniae* in exposed controls, which was detected 25% less frequently compared to nonexposed controls. Bacterial detection in induced sputum by PCR decreased 7% for exposed compared to nonexposed cases. For every additional 1 mL of blood culture specimen collected, microbial yield increased 0.51% (95% confidence interval, 0.47%–0.54%), from 2% when volume was ≤1 mL to approximately 6% for ≥3 mL.

**Conclusions.:**

Antibiotic exposure and blood culture volume affect detection of bacterial pathogens in children with pneumonia and should be accounted for in studies of etiology and in clinical management.

Identifying the microbiological cause of pneumonia can help guide treatment and prevention strategies. However, this remains challenging for a variety of reasons, including suboptimal diagnostic accuracy of testing methods, difficulties obtaining adequate blood specimen volumes in young children, and the administration of antibiotics prior to collection of diagnostic specimens [1–3]. Treatment with antibiotics prior to specimen collection has been shown to decrease detection of potential bacterial pathogens from sterile body fluids in children by culture and, to a lesser extent, by nucleic acid detection methods [4–8]. Less is known about the effect of antibiotic treatment on bacterial pathogen detection from nonsterile respiratory specimens, such as nasopharyngeal-oropharyngeal (NP/OP) swabs.

Blood volume has been reported as the most important factor influencing the sensitivity of blood cultures among patients with sepsis [3, 9], and each additional milliliter of blood cultured has been associated with an increased microbial yield of up to 3% per specimen for adults [10]. Volume was previously assumed to be less important for children given the higher density of microorganisms typically found in their blood, but recent studies have confirmed that low-level bacteremia is more common than previously thought in pediatric patients [3], and increasing blood volume in children has been shown in several studies to increase bacterial yield [6, 11–14].

Here we aim to quantify the effect of antibiotic exposure and specimen volume on bacterial pathogen recovery from blood and respiratory specimens taken from children hospitalized with severe and very severe pneumonia, and to investigate the effect of key covariates of interest including age, human immunodeficiency virus (HIV) infection status, chest radiograph (CXR) finding, disease severity, and research site on these estimates.

## METHODS

The Pneumonia Etiology Research for Child Health (PERCH) study was a multicountry, case-control study of the etiology of severe pneumonia in children from developing countries [15]. Enrollment occurred for 24 months at each of 9 study sites located in 7 countries: Dhaka and Matlab, Bangladesh; Basse, The Gambia; Kilifi, Kenya; Bamako, Mali; Soweto, South Africa; Nakhon Phanom and Sa Kaeo, Thailand; and Lusaka, Zambia. Identification and selection of cases and controls have been described previously [16]. In brief, cases were hospitalized children aged 1–59 months with World Health Organization–defined severe or very severe pneumonia [17]. Controls were randomly selected from the community and were frequency-matched to cases on month of enrollment and by the following age groups: 1–5 months, 6–11 months, 12–23 months, and 24–59 months. A second set of controls was frequency matched to HIV-infected cases at the 2 sites (Zambia and South Africa) with high HIV prevalence. Controls with respiratory tract illness were included only if they did not have case-defining severe or very severe pneumonia.

Blood, nasopharyngeal, and oropharyngeal (NP/OP) swabs, induced sputum (IS), and urine were collected from cases at study enrollment. A CXR was collected from cases as soon as possible after clinical evaluation and study enrollment and was reviewed by a trained panel using standardized guidelines for interpretation [18]. CXRs were considered normal if there was no consolidation or infiltrate present. Blood, NP/OP swabs, and urine were collected from controls at enrollment.

The study used standard culture methods to detect putative pneumonia pathogens from blood and respiratory specimens for the purpose of ascribing etiology [19]. A minimum 2-mL blood specimen was requested for culture for all participants weighing ≥3 kg, with a target volume of 3 mL. Culture bottles were weighed before and after inoculation, with the difference in weight multiplied by 1.05 (density of blood) to determine specimen volume in milliliters [20]. In the absence of strong evidence otherwise, organisms cultured from blood that met the a priori contaminant definition were considered as contaminants [19], and the identification of noncontaminant blood culture isolates was confirmed at the study reference laboratory. Enrollment blood specimens from cases and controls were also tested for *Streptococcus pneumoniae* by polymerase chain reaction (PCR). Respiratory specimens were tested by multiplex PCR including 33 different bacterial and viral targets [19].

To detect exposure to antibiotics, a serum bioassay was performed on sera collected from all cases and controls at enrollment. A 6-mm filter paper disc was inoculated with 20 µL of serum and then placed on a Mueller-Hinton plate seeded with a 0.5 McFarland suspension of a sensitive *Staphylococcus aureus* strain (ATCC 25923). Any zone of inhibited bacterial growth around the disc after 18–24 hours of incubation was measured and recorded as evidence of serum antibiotic activity. Using urine, the same bioassay method was applied for a subset of cases in South Africa.

Study clinicians were encouraged to collect diagnostic specimens prior to antibiotic administration whenever possible. Clinicians recorded the time of first antibiotic administration at the study facility and whether specimens had already been collected; additionally, the time and date of specimen collection and antibiotic administration were recorded. If the child was referred from another facility to the PERCH study facility, the parents were asked whether the child had received antibiotics at the referring facility. In some cases, documentation of the administration of antibiotics accompanied the child. Parents were also asked whether their children received antibiotics within the past 48 hours, or were receiving cotrimoxazole prophylaxis, but these self-reported data were not believed to be consistently reliable, were not good predictors of serum antimicrobial activity, and were therefore not included in the antibiotic exposure definition.

Cases were defined as having “antibiotic exposure prior to specimen collection” if they met any of the following criteria: had a positive serum antibiotic bioassay, received antibiotics at a referral facility (based on parental report or documentation), or received antibiotics at the study facility prior to the collection of specimens (assessed by clinician report and time/date calculation). Controls were considered antibiotic exposed if they had a positive serum bioassay. Cases and controls whose results were negative or not applicable for all of the exposure indicators were considered to not have antibiotic exposure. Those who did not meet any of the exposure criteria but had missing data for 1 or more indicators were considered to have unknown exposure status.

### Statistical Methods

Comparisons of binary outcomes were made using χ^2^ or Fisher exact test. Serum and urine antibiotic bioassay results were compared using McNemar paired χ^2^ test. Multiple logistic regression and linear regression were used for adjusted analyses. Adjusted odds ratios (ORs) for laboratory outcomes in cases included site, age category, pneumonia severity, CXR status, and when relevant, blood culture volume, as covariates. Adjusted odds ratios for laboratory outcomes in controls included site, age category, and HIV status as covariates. Percentage reduction was estimated using 1 minus the relative risk, which was calculated using the odds ratios. All *P* values are 2-sided. All analyses were done using STATA software version 13.0.

### Ethical Considerations

The PERCH study protocol was approved by the institutional review board or ethical review committee at each of the study site institutions and at the Johns Hopkins Bloomberg School of Public Health. Parents or guardians of all participants provided written informed consent.

## RESULTS

Between August 2011 and January 2014, PERCH enrolled 4232 children hospitalized with severe (n = 2862) or very severe (n = 1370) pneumonia and 5325 community controls. Nearly all (96.4%) cases received antibiotics at admission to the PERCH study facilities. The most commonly prescribed antibiotics at admission, either alone or in combination, were amoxicillin (1909/4709 [46.8%]), an aminoglycoside (2023 [49.6%]), and penicillin (1401 [34.3%]). Nearly half of cases (1833 [44.9%]) received combination therapy containing aminoglycoside with amoxicillin and/or penicillin. Among the 464 treated cases who did not receive amoxicillin or penicillin, 410 (88.4%) received ceftriaxone instead. With the exception of amoxicillin and amoxicillin/clavulanate, antibiotics were administered parenterally >95% of the time. Routine cotrimoxazole prophylaxis was reported in 79 of 902 (8.8%) cases and 138 of 940 (14.7%) controls in South Africa, and 76 of 611 (12.4%) of cases and 139 of 686 (20.3%) controls in Zambia, the 2 sites with highest HIV prevalence.

### Antibiotic Exposure Prior to Specimen Collection

Among cases, 18.3% of 4211 blood specimens, 25.3% of 4223 NP/OP swabs, 71.7% of 3800 IS specimens, and 68.6% of 2388 urine specimens were collected after antibiotic administration at the study facility. Blood was the first specimen obtained for 91.3% of 4176 cases who had respiratory or urine samples collected in addition to blood. The proportion of blood samples collected after antibiotic administration at the study facility varied by site, from 4.3% in Mali to 47.9% in Zambia (Table 1).

**Table 1.** Frequency of Blood Specimens Collected After Prior Exposure to Antibiotics Among Cases Aged 1–59 Months Hospitalized With Pneumonia and Community Controls Enrolled in the Pneumonia Etiology Research for Child Health (PERCH) Study

Site	A. Antibiotics Administered at Study Facility, Before Blood Collection		B. Antibiotics Administered at Referral Facility		C. Antibiotics Administered at Any Facility Before Blood Collection (A or B)		D. Serum Antibiotic Activity				Antibiotics Received Before Blood Collection (Any of A, B, or D for Cases and D Only for Controls)			
	Cases		Cases		Cases		Cases		Controls		Cases		Controls	
All sites	4211^a^	769 (18.3)	4232	621 (14.7)	4232	1054 (24.9)	3995	1022 (25.6)	4864	110 (2.3)	4065	1767 (43.5)	4864	110 (2.3)
Kenya	634	183 (28.9)	634	0 (0.0)	634	183 (28.9)	562	73 (13.0)	777	22 (2.8)	585	238 (40.7)	777	22 (2.8)
The Gambia	622	33 (5.3)	638	8 (1.3)	638	41 (6.4)	579	43 (7.4)	616	1 (0.2)	571	78 (13.7)	616	1 (0.2)
Mali	674	29 (4.3)	674	80 (11.9)	674	105 (15.6)	672	129 (19.2)	700	19 (2.7)	670	174 (26.0)	700	19 (2.7)
Zambia														
HIV infected	103	36 (35.0)	100	82 (82.0)	103	89 (86.4)	99	36 (36.4)	75	8 (10.7)	101	90 (89.1)	75	8 (10.7)
HIV uninfected	513	259 (50.5)	502	421 (83.9)	513	461 (89.9)	490	141 (28.8)	533	21 (3.9)	501	464 (92.6)	533	21 (3.9)
South Africa														
HIV infected	115	16 (13.9)	115	0 (0.0)	115	16 (13.9)	110	64 (58.2)	118	16 (13.6)	105	69 (65.7)	118	16 (13.6)
HIV uninfected	805	119 (14.8)	805	0 (0.0)	805	119 (14.8)	768	375 (48.8)	764	8 (1.1)	719	411 (57.2)	764	8 (1.1)
Bangladesh	522	66 (12.6)	525	0 (0.0)	522	66 (12.6)	493	118 (23.9)	713	10 (1.4)	517	151 (29.2)	713	10 (1.4)
Thailand	223	28 (12.6)	222	43 (19.4)	223	51 (22.9)	222	43 (19.4)	568	5 (0.9)	223	69 (30.9)	568	5 (0.9)

Data are presented as No. (%).

Abbreviation: HIV, human immunodeficiency virus.

^a^Numbers for all sites exclude cases and controls with missing antibiotic exposure status (see Supplementary Table 1).

There was significant variation in the proportion of children receiving antibiotics at referral facilities prior to PERCH enrollment. Three sites (Bangladesh, Kenya, and South Africa) had no facility that formally referred cases. In Zambia, 92.7% of cases visited another facility before coming to the study hospital and 83.6% received antibiotics at that facility. At the 3 remaining sites, 1.3% (The Gambia), 11.9% (Mali), and 19.4% (Thailand) of cases received antibiotics at a referral facility (Table 1).

Serum antibiotic bioassay was performed on 94.1% of cases and 90.6% of controls; antibiotics were detected in 25.6% and 2.3%, respectively, with the highest frequency of detection in South African cases (50.0%) (Table 1). Paired serum and urine bioassay results were compared in a subset of 476 participants (246 cases, 230 controls) from South Africa. Of these, 117 (24.6%) serum specimens and 200 (42.0%) urine specimens were bioassay positive. Treating the urine bioassay as the reference standard, the sensitivity of the serum bioassay was 111 of 200 (56%).

Of the 96.0% of cases and 91.3% of controls (range across sites, 90%–99% and 86%–96%, respectively; Supplementary Table 1) with data available to define “antibiotic exposure” prior to blood collection, 43.5% of cases and 2.3% of controls met the composite exposure definition (range across sites, 13.7%–92.0% and 0.2%–4.8%, respectively; Table 1). NP/OP, IS, and urine specimens met the composite definition for antibiotic exposure in 48.8%, 80.4%, and 81.7% of cases, with variation by site (Table 2). Among cases, younger age (<12 months), an abnormal CXR (consolidation or infiltrate), and very severe (compared to severe) pneumonia were associated with antibiotic exposure prior to specimen collection (Table 3). Among controls, HIV infection was associated with increased odds of antibiotic exposure in South Africa and Zambia (Table 3).

**Table 2.** Respiratory and Urine Specimens Collected After Exposure to Antibiotics Among Children Aged 1–59 Months Hospitalized With Pneumonia

Site	NP/OP Swabs		Induced Sputum		Urine	
	No.^a^	Antibiotic Exposed^b^, No. (%)	No.^a^	Antibiotic Exposed^b^, No. (%)	No.^a^	Antibiotic Exposed^b^, No. (%)
All sites	4089	1994 (48.8)	3826	3075 (80.4)	3752	3066 (81.7)
Kenya	613	392 (40.7)	573	550 (96.0)	568	489 (86.1)
Gambia	578	89 (15.4)	556	134 (24.1)	586	402 (68.6)
Mali	670	176 (26.3)	558	467 (83.7)	522	453 (86.8)
Zambia						
HIV infected	102	91 (89.2)	98	98 (100.0)	101	100 (99.0)
HIV uninfected	509	467 (91.8)	496	496 (100.0)	484	483 (99.8)
South Africa						
HIV infected	111	76 (68.5)	109	101 (92.7)	107	92 (86.0)
HIV uninfected	784	474 (60.4)	770	719 (93.4)	689	545 (79.1)
Bangladesh	497	134 (27.0)	481	336 (69.9)	488	324 (66.4)
Thailand	223	94 (42.2)	185	174 (94.1)	206	177 (85.9)

Abbreviations: HIV, human immunodeficiency virus; NP/OP, nasopharyngeal/ oropharyngeal.

^a^Of those with specimen collected and known antibiotic administration status.^b^Antibiotic exposure defined as any of the following: having a positive serum bioassay, received antibiotics at a referral facility, or received antibiotics at the study facility prior to the collection of enrollment blood specimen (assessed by clinician report and time/date calculation).

**Table 3.** Association of Hospitalized Case and Community Control Characteristics With Antibiotic Exposure^a^ Prior to Blood Collection

	Cases		Controls	
	Antibiotic Exposure, No.^b^ (%)	AOR^c^ (95% CI)	Antibiotic Exposure, No.^b^ (%)	AOR^c^ (95% CI)
Overall	1767 (43.5)		110 (2.3)	
Age, mo				
1–5	768 (48.0)	Ref	38 (2.6)	Ref
6–11	421 (48.2)	1.00 (.85–1.19)	28 (2.4)	0.95 (.58–1.55)
12–23	309 (36.1)	**0.61 (.51**–**.72**)	29 (2.4)	0.93 (.57–1.52)
24–59	179 (34.8)	**0.58 (.47**–**.71**)	15 (1.5)	0.56 (.31–1.03)
*P* value for variation		**<.001**		.10
HIV status^d^				
Negative/unknown	875 (71.7)	Ref	29 (2.2)	Ref
Positive	159 (77.2)	1.33 (.94–1.89)	24 (12.4)	**6.21 (3.53**–**10.92**)
Chest radiograph findings				
Normal	547 (37.6)	Ref	…	…
Abnormal	801 (44.8)	**1.35 (1.17**–**1.55**)		
Pneumonia severity			…	…
Severe	1,044 (40.1)	Ref		
Very severe	633 (51.1)	**1.57 (1.37**–**1.79**)		

Abbreviations: AOR, adjusted odds ratio; CI, confidence interval; HIV, human immunodeficiency virus. Bold values are statistically significant at α = .05 level.

^a^Antibiotic exposure defined for cases as meeting any of the following criteria: having a positive serum bioassay, received antibiotics at a referral facility, or administered antibiotics at the study facility prior to the collection of specimens (assessed by clinician report and time/date calculation); for controls as having a positive serum bioassay. Participants with unknown antibiotic exposure data are excluded from these analyses.

^b^Numbers for all sites exclude cases and controls with missing antibiotic exposure status (see Supplementary Table 1).

^c^Odds ratios for cases are adjusted for age category, chest radiograph status, pneumonia severity, and study site as well as HIV status for relevant study sites (South Africa and Zambia only). Odds ratios for controls are adjusted for age category, HIV status, and research site.

^d^Restricted to sites with HIV prevalence >1.0% among cases (Zambia and South Africa).

### Antibiotic Exposure and Detection of Organisms by Culture

The isolation of a noncontaminant organism from blood was significantly less frequent in cases exposed to antibiotics before specimen collection compared to those who received antibiotics after specimen collection (adjusted OR, 0.54; 95% confidence interval [CI], .29–.98) after adjusting for site, blood volume, age category, abnormal CXR, and very severe (compared to severe) pneumonia (Table 4). Associations did not vary significantly by bacteria (Table 4). Trends were consistent when limited to the subgroup of CXR-positive, HIV-uninfected cases, but did not reach statistical significance (Supplementary Table 2).

**Table 4.** Association of Antibiotic Exposure With Detection of Bacterial Organisms by Culture Among Participants Enrolled in the Pneumonia Etiology Research for Child Health (PERCH) Study

Specimen	Antibiotic-Pretreated^a^	Bacteria Isolated, No. (%)	Adjusted OR^b^ (95% CI)
Blood (cases)		Any bacteria^c^	
	Yes (n = 1677)	61 (3.6)	**0.54** ^**d**^ **(.29–.98)**
	No (n = 2167)	75 (3.5)	
		Any gram-positive bacteria	
	Yes (n = 1636)	10 (1.2)	**0.26 (.10–.72**)
	No (n = 2092)	42 (2.0)	
		*Streptococcus pneumoniae*	
	Yes (n = 1625)	9 (0.6)	**0.20 (.06–.70**)
	No (n = 2125)	33 (1.6)	
		*Staphylococcus aureus*	
	Yes (n = 1624)	8 (0.5)	0.36 (.03**–**4.33)
	No (n = 2099)	7 (0.3)	
		Any gram-negative bacteria	
	Yes (n = 1656)	40 (2.4)	0.88 (.41**–**1.87)
	No (n = 2124)	32 (1.5)	
		*Hemophilus influenzae*	
	Yes (n = 1623)	7 (0.4)	**0.14 (.02–.99**)
	No (n = 2092)	13 (0.6)	
		Enterobacteriaceae	
	Yes (n = 1626)	10 (0.6)	1.22 (.29**–**5.04)
	No (n = 2100)	8 (0.4)	
		*Salmonella* spp	
	Yes (n = 1630)	14 (0.9)	2.89 (.62**–**13.46)
	No (n = 2096)	4 (0.2)	
		Nonfermenting gram-negative rods	
	Yes (n = 1625)	9 (0.6)	8.61 (.61**–**122.06)
	No (n = 2095)	3 (0.1)	
NP (cases)		*S. pneumoniae*	
	Yes (n = 1924)	756 (39.3)	**0.47 (.40–.56**)
	No (n = 1973)	1264 (64.1)	
NP (controls)		*S. pneumoniae*	
	Yes (n = 103)	48 (46.6)	**0.39 (.26–.58**)
	No (n = 4712)	3290 (69.8)	
IS (cases)^e^		Any bacteria, excluding normal oropharyngeal flora	
	Yes (n = 2844)	1974 (68.5)	**0.24 (.17–.33**)
	No (n = 751)	697 (92.8)	
		*S. pneumoniae*	
	Yes (n = 1444)	547 (37.9)	**0.14 (.10–.20**)
	No (n = 511)	457 (89.4)	
		*H. influenzae*	
	Yes (n = 1713)	816 (47.6)	**0.17 (.12–.24**)
	No (n = 555)	501 (90.3)	
		*Moraxella catarrhalis*	
	Yes (n = 1543)	646 (41.9)	**0.21 (.13–.33**)
	No (n = 369)	315 (85.4)	
		*S. aureus*	
	Yes (n = 1201)	304 (25.3)	**0.31 (.19–.50**)
	No (n = 122)	68 (55.7)	
		Other gram-negative rods^f^	
	Yes (n = 897)	0 (0.0)	…
	No (n = 54)	0 (0.0)	

Abbreviations: CI, confidence interval; IS, induced sputum; NP, nasopharyngeal; OR, odds ratio. Bold values are statistically significant at α = .05 level.

^a^Antibiotic exposure defined for cases as meeting any of the following criteria: having a positive serum bioassay, received antibiotics at a referral facility, or administered antibiotics at the study facility prior to the collection of specimens (assessed by clinician report and time/date calculation); for controls as having a positive serum bioassay. Participants with unknown antibiotic exposure data are excluded from these analyses.

^b^Relative odds of culture positivity (vs no growth of any organism) given antibiotic exposure, compared to those who are not pretreated. Odds ratios were adjusted for study site, pneumonia severity, chest radiograph (CXR) finding, blood culture specimen volume >3 mL, and age category. Respiratory specimens (NP, IS) in cases were adjusted for the same variables with the exception of blood specimen volume; NP in controls was adjusted for study site, age, and human immunodeficiency virus status.

^c^Excluding contaminants.

^d^The crude odds ratio for the detection of any noncontaminant bacteria blood pathogen (antibiotic exposed vs unexposed) was 1.05 (95% CI, .75–1.49) due to a higher proportion of blood culture–positive specimens among the subset of cases with missing or uninterpretable CXR findings. After adjusting for CXR status, the OR is 0.92 (95% CI, .63–1.35). Further adjusting for, age, study site, pneumonia severity, and blood volume >3 mL produced the adjusted OR shown here.

^e^No quality stringency criteria applied.

^f^Other gram-negative rods isolated from induced sputum includes *Acinetobacter* spp, *Enterobacter aerogenes*, *Enterobacter cloacae*, *Pseudomonas* species, and mixed gram-negative rods.

The culture yield of *S. pneumoniae* from NP swabs was significantly reduced for both cases and controls pretreated with antibiotics, and this relationship was consistent when restricted to the CXR-positive, HIV-uninfected cases and HIV-uninfected controls (Table 4; Supplementary Table 2). Among IS specimens collected from cases, antibiotic exposure was associated with reduced odds of growth of any isolate (Table 4). Among CXR-positive, HIV-uninfected cases, the relationship was the same (Supplementary Table 2).

### Antibiotic Exposure and Detection of Organisms by PCR

Detection of pneumococcus in whole blood by PCR was not significantly affected by antibiotic exposure (Table 5; Supplementary Table 3). For NP/OP specimens tested by Fast-track PCR, the mean number of positive bacterial targets detected in case specimens was higher in those with antibiotic exposure compared to those without (2.21 vs 2.05; *P* < .001); this did not differ by CXR or HIV status. In contrast, controls that were antibiotic exposed had fewer bacterial targets detected by NP/OP PCR than unexposed controls (1.18 vs 2.21; *P* < .001), which also did not differ by HIV status. Cases and controls with antibiotic exposure before specimen collection were less likely to be positive by NP/OP PCR for *S. pneumoniae*, *Haemophilus influenzae*, or *Moraxella catarrhalis* (Table 5; Supplementary Table 3). For cases with NP samples positive by PCR for any of *S. pneumoniae*, *H. influenzae*, or *M. catarrhalis*, bacterial density was lower for those who were pretreated compared to those who were not (Table 5; Supplementary Table 3). Cases were also more likely to have a *S. pneumoniae* PCR result above the density threshold (6.9 log_10_ copies/mL) associated with microbiologically confirmed pneumococcal pneumonia threshold [21] if they were not exposed to antibiotics prior to specimen collection compared to those who were exposed. Trends were generally similar in controls, and when limited to the CXR-positive, HIV-uninfected cases and HIV-uninfected controls (Table 5; Supplementary Table 3). PCR detection of any bacteria, *S. pneumoniae*, *H. influenzae*, and *M. catarrhalis* in IS specimens was significantly lower in exposed cases (Table 5). The same was true for CXR-positive, HIV-uninfected cases, except *S. pneumoniae* did not reach statistical significance in this group (Supplementary Table 3). Among cases with IS positive for *S. pneumoniae*, *H. influenzae*, or *M. catarrhalis* by PCR, reduced bacterial density was associated with antibiotic exposure (Table 5; Supplementary Table 3).

**Table 5.** Association of Polymerase Chain Reaction Outcome by Antibiotic Exposure Status Among Children Aged 1–59 Months Enrolled in the Pneumonia Etiology Research for Child Health (PERCH) Study

Antibiotic- Pretreated^a^	PCR Positive, No. (%)	Adjusted OR^b^ (95% CI)	Mean (SD) Density (Log_10_ Copies/mL) Among Positives	Fold Difference^c^ (95% CI)
Whole blood pneumococcal PCR—cases				
Yes (n = 1559)	113 (7.3)	1.10 (.79–1.54)	2.81 (0.96)	0.13 (–.14 to .39)
No (n = 2122)	148 (7.0)		2.76 (0.98)	
Whole blood pneumococcal PCR—controls				
Yes (n = 106)	9 (8.5)	1.44 (.71–2.92)	2.58 (0.75)	0.22 (–.20 to .64)
No (n = 4596)	254 (5.5)		2.31 (0.61)	
NP/OP respiratory PCR—cases				
	Any bacteria			
Yes (n = 1894)	1747 (92.2)	**0.74 (.56**–**.98**)	…	…
No (n = 2022)	1896 (93.8)			
	*Streptococcus pneumoniae*			
Yes (n = 1892)	1321 (69.8)	**0.83 (.70**–**.98**)	5.39 (1.27)	**–0.40 (–.52 to –.29**)
No (n = 2021)	1502 (74.3)		5.94 (1.27)	
	*S. pneumoniae* above threshold^d^			
Yes (n = 1892)	144 (7.6)	**0.43 (.33**–**.55**)	7.33 (0.33)	0.06 (**–**.03 to .15)
No (n = 2021)	359 (17.8)		7.34 (0.35)	
	*Haemophilus influenzae*			
Yes (n = 1892)	917 (48.5)	**0.84 (.71**–**.99**)	5.62 (1.24)	**–0.28 (–.41 to –.16**)
No (n = 2018)	1186 (58.8)		5.93 (1.20)	
	*Moraxella catarrhalis*			
Yes (n = 1892)	1223 (64.6)	**0.83 (.70**–**.99**)	5.17 (1.23)	**–0.32 (–.44 to –.21**)
No (n = 2018)	1372 (68.0)		5.59 (1.12)	
	*Staphylococcus aureus*			
Yes (n = 1892)	349 (18.5)	0.93 (.74–1.15)	5.34 (1.17)	5.34 (1.17)
No (n = 2021)	329 (16.3)		5.34 (1.17)	
NP/OP respiratory PCR—controls				
	Any bacteria			
Yes (n = 106)	91 (85.9)	**0.40 (.22**–**.74**)	…	…
No (n = 4596)	4398 (95.7)			
	*S. pneumoniae*			
Yes (n = 106)	61 (57.6)	**0.40 (.26**–**.59**)	5.42 (1.20)	**–**0.19 (**–**.46 to .08)
No (n = 4594)	3561 (77.5)		5.63 (1.11)	
	*S. pneumoniae* above threshold^d^			
Yes (n = 106)	106 (3.8)	0.44 (.16–1.23)	7.14 (0.15)	**–**0.19 (**–**.46 to .08)
No (n = 4594)	365 (8.0)		7.25 (0.29)	
	*H. influenzae*			
Yes (n = 106)	49 (46.2)	0.98 (.66–1.46)	5.30 (1.09)	**–**0.19 (**–**.48 to .10)
No (n = 4589)	2391 (52.1)		5.60 (1.05)	
	*M. catarrhalis*			
Yes (n = 106)	62 (58.5)	**0.44 (.30**–**.67**)	5.09 (1.33)	**–0.27 (–.52 to –.02**)
No (n = 4589)	3416 (74.4)		5.48 (1.01)	
	*S. aureus*			
Yes (n = 106)	15 (14.2)	1.08 (.61–1.02)	5.18 (1.21)	0.03 (–.50 to .55)
No (n = 4594)	623 (13.6)		5.00 (1.04)	
Induced sputum PCR—cases				
	Any bacteria			
Yes (n = 2791)	2496 (89.3)	**0.52 (.32**–**.83**)	…	…
No (n = 717)	688 (96.0)			
	*S. pneumoniae*			
Yes (n = 2791)	1857 (66.5)	**0.68 (.52**–**.89**)	5.02 (1.18)	**–0.23 (–.35 to –.11**)
No (n = 717)	559 (78.0)		6.15 (1.09)	
	*H. influenzae*			
Yes (n = 2790)	1311 (47.0)	**0.67 (.52**–**.85**)	5.24 (1.16)	**–0.30 (–.44 to –.17**)
No (n = 717)	497 (69.3)		6.05 (1.13)	
	*M. catarrhalis*			
Yes (n = 2790)	1560 (55.9)	**0.56 (.44**–**.72**)	4.86 (1.30)	**–0.32 (–.46 to –.18**)
No (n = 717)	538 (75.0)		5.86 (1.18)	
	*S. aureus*			
Yes (n = 2791)	447 (16.0)	0.87 (.63–1.22)	5.01 (1.03)	**–**0.07 (**–**.30 to .16)
No (n = 717)	99 (13.8)		5.23 (1.07)	

Abbreviations: CI, confidence interval; NP/OP, nasopharyngeal/oropharyngeal; OR, odds ratio; PCR, polymerase chain reaction; SD, standard deviation. Bold values are statistically significant at α = .05 level.

^a^Antibiotic exposure defined for cases as any of the following: having a positive serum bioassay, received antibiotics at a referral facility, or received antibiotics at the study facility prior to the collection of specimens (assessed by clinician report and time/date calculation); Antibiotic exposure for controls is defined as having a positive serum bioassay. Participants with unknown antibiotic exposure status are excluded from analyses.

^b^Relative odds of pathogen detection given antibiotic exposure, compared to those who were not pretreated. Odds ratios are adjusted for study site, pneumonia severity, chest radiograph status, and age. NP/OP outcomes in controls are adjusted for study site, human immunodeficiency virus infection status, and age.

^c^Fold-difference in mean log_10_ transformed PCR density (copies/mL) for pretreated compared to non-pretreated individuals, among those PCR positive.

^d^Above PCR density threshold (6.9 log_10_ copies/mL) associated with case status [21].

### Blood Volume and Detection of Organisms by Blood Culture

**Figure 1.**
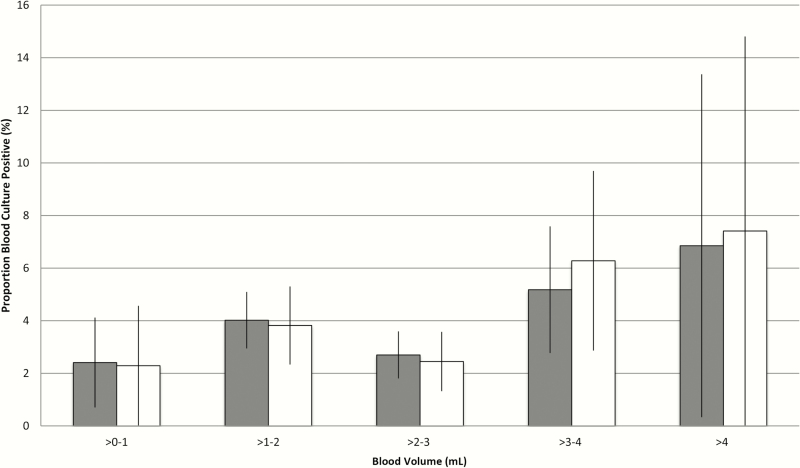
Blood culture positivity (excluding contaminant organisms) by specimen volume, with 95% confidence intervals. Solid bars: all cases. White bars: all cases without antibiotic exposure prior to blood specimen collection.

Blood culture volumes were available for 3553 of 4232 (84.0%) specimens. Of the 679 specimens with missing volumes, 558 (82.2%) were from South Africa. Mean blood culture volume was 2.05 mL (standard deviation [SD], 0.85 mL); only 12.4% of all blood culture specimens met the target volume of ≥3.0 mL (site range, 2.8% in Bangladesh to 33.1% in Mali; Table 6). Older age was associated with increased probability of a blood culture specimen volume ≥3.0 mL in The Gambia and Thailand (Table 6). An increase in blood culture yield was observed with greater blood culture volumes, from approximately 2% among specimens with ≤1 mL to >6% among specimens with >4 mL (Figure 1). In a univariate linear regression model, each additional 1 mL of blood collected was associated with a 0.51% (95% CI, 0.47%–0.54%) absolute increase in blood culture yield. Specimens with volume of at least 3 mL were significantly more likely to yield a blood culture pathogen compared to those with a volume of ≤1 mL (adjusted OR, 4.85; 95% CI, 1.24–18.99) after adjusting for site, age, CXR status, severity, and antibiotic exposure.

**Table 6.** Association of Blood Culture Volumes ≥3 mL With Age and Site among Children Aged 1–59 Months Hospitalized With Pneumonia

	Kenya (n = 632^a^)		Gambia (n = 579)		Mali (n = 661)		Zambia (n = 596)		South Africa (n = 362)		Thailand (n = 499)		Bangladesh (n = 224)	
Volume, mL, median (IQR)	1.70 (1.13–2.26)		2.17 (1.70–2.55)		2.36 (1.70–3.30)		1.95 (1.55–2.38)		1.13 (0.85–1.79)		2.41 (1.84–2.93)		2.06 (1.94–2.92)	
Volume ≥3 mL, No. (%)	41 (4.9)		67 (11.6)		219 (33.1)		44 (7.4)		15 (4.1)		50 (22.3)		14 (2.8)	
	No.^b^ (%)	AOR^c^ (95% CI)	No. (%)	AOR (95% CI)	No. (%)	AOR (95% CI)	No. (%)	AOR (95% CI)	No. (%)	AOR (95% CI)	No. (%)	AOR (95% CI)	No. (%)	AOR (95% CI)
Age, mo														
1–5 (ref)	6 (2.9)		14 (6.0)		100 (33.8)		21 (6.7)		8 (4.6)		6 (15.8)		3 (2.4)	
6–11	7 (5.4)	1.93 (.63–5.88)	21 (16.0)	**3.0** **(1.5–6.13)**	46 (30.5)	0.86 (.56–1.31)	11 (7.8)	1.18 (.55–2.52)	2 (2.1)	0.45 (.09–2.18)	6 (12.0)	0.73 (.21–2.46)	3 (2.7)	1.11 (.22–5.61)
12–23	11 (6.4)	2.31 (.84–6.39)	22 (17.9)	**3.42** **(1.68–6.13)**	47 (33.3)	0.98, (.64–1.50)	9 (10.3)	1.62 (.71–3.68)	2 (3.3)	0.72 (.15–3.49)	15 (21.7)	1.48 (.52–4.20)	5 (2.9)	**1.22** **(.29–5.19)**
24–59	7 (5.7)	2.01 (.66–6.14)	10 (11.0)	**1.94** **(.83–4.54)**	26 (35.6)	1.08 (.63–1.85)	3 (5.9)	0.88 (.25–3.06)	3 (9.1)	2.09 (.52–8.32)	24 (33.3)	**2.79 (1.01–7.63**)	3 (3.4)	1.42 (.28–7.20)
*P* value for variation		.16		**.02**		.88		.59		.59		**<.01**		**.66**

Abbreviations: AOR, adjusted odds ratio; CI, confidence interval; IQR, interquartile range. Bold values are statistically significant at α = .05 level.

^a^Numbers for all study sites exclude cases and controls with missing antibiotic exposure status (see Supplementary Table 1).

^b^Number (percentage) with blood culture specimen volume ≥3 mL.

^c^Relative odds of having a blood culture specimen volume ≥3 mL, adjusted for chest radiograph status, pneumonia severity, and study site.

## DISCUSSION

To our knowledge, this is the first study to evaluate the effect of antibiotic exposure across a range of specimen types and bacterial pathogens in a population of children hospitalized with severe or very severe pneumonia. Among cases and controls, exposure reduced the detection of common pneumonia bacteria by culture and, to a lesser extent, PCR. After adjusting for site and other relevant covariates, antibiotic exposure reduced blood culture yield of bacteria by approximately 45%. Results were similar for CXR-positive and HIV-uninfected cases and controls. Antibiotic exposure before specimen collection among hospitalized children was common in our study, with variation by study site and specimen type. Blood was almost always the first specimen collected and was collected after exposure to antibiotics in approximately 40% of cases, while approximately 50%–80% of respiratory and urine specimens were collected after cases received antibiotics. Antibiotic use among community controls was very low, with the exception of HIV-infected controls receiving routine cotrimoxazole prophylaxis. We also observed that increasing blood volume was associated with greater recovery of blood pathogens by culture.

Our findings are consistent with a study from rural Kenya that found that recent antibiotic use (measured by plasma microbial activity) in children <13 years of age reduced blood culture yield by 62%–73% in patients with severe or fatal disease, and greater blood volume yielded higher bacterial detection by culture [6]. Similarly in Thailand, incidence rates of hospitalized pneumococcal bacteremia among children aged <5 years were 63% higher when adjusted for antibiotic use defined by serum antimicrobial activity [4]. *Staphylococcus aureus*, Enterobacteriaceae, and nonfermenting gram-negative rods were not affected by antibiotic exposure in our study, which is expected given that most first-line antibiotics do not have activity against these classes of bacteria.

For NP culture specimens, antibiotic exposure reduced detection of pneumococcus by culture by approximately 30% in cases and controls, with a more modest reduction in detection (by approximately 5%–7%) of bacterial pathogens by PCR. These findings are similar to those in a study of an elderly population in Finland where detection of pneumococcus was significantly reduced for NP culture and showed a nonsignificant trend toward reduction by PCR [22]. The reduction in detection by PCR attributable to antibiotic exposure was greater for controls than cases, which could be due to the lower bacterial densities in controls. On average, IS specimens were collected several hours and up to a day following blood and NP/OP swabs, which could explain the greater effect of antibiotic exposure on detection of pathogens in IS, particularly for children whose source of antibiotic exposure occurred at hospital admission.

Our analyses have several limitations. Most importantly, there was potential for misclassification of antibiotic exposure status. Antibiotic type, dose, and timing relative to specimen collection could all be expected to impact serum antimicrobial activity, potentially resulting in misclassification, and could not be taken into account in our definition. Although the timing between administration and specimen collection at the study facility was available, we felt that the application of a time cutoff for exposure would be arbitrary and therefore considered any specimen that was collected following antibiotic administration to be exposed. Serum bioassay was selected over urine to evaluate presence of antibiotics because urine was >4 times more likely than serum to be collected after antibiotic administration. However, serum has been shown to be less sensitive than urine for this application [23–25], and the 56% sensitivity we observed for serum compared to urine is the same as reported elsewhere [23]. Another weakness of the serum bioassay is that given the half-life of antibiotics in the blood, it may only reliably detect antimicrobial exposure within the past 8 hours [26] and, as a result, receipt of antibiotics prior to this time frame may result in sterilization of the blood while being undetectable. In addition to these limitations, it is possible that there was selection bias for antibiotic exposure in that cases with bacterial pneumonia may have been more ill and therefore more likely to have received antibiotic treatment prior to admission. If so, our estimates of the effect of antibiotic exposure on bacterial recovery would be underestimated.

Specimens identified as “not exposed to antibiotics” may also have been misclassified as antibiotics administered by the parents were not captured in our definition. If so, our findings would again be biased toward the null, and the true impact of exposure would therefore be of a greater magnitude than what is reported here. The level of misclassification might vary by study site, which may explain some site differences, as might the exclusion of participants missing antibiotic exposure status, which also varied by site. Finally, small numbers of positive blood cultures limited our ability to analyze pathogen-specific associations with antibiotic exposure, and the large proportion of missing blood culture volumes from South Africa reduced the precision of the estimate of the impact of blood culture volume on positivity for this site.

Despite these limitations, our data reveal consistent and statistically significant reductions in the detection of bacterial pathogens in blood and respiratory specimens of children associated with exposure to antibiotics prior to specimen collection and low blood volume. Because few bacterial pneumonias are bacteremic, a reduction in the sensitivity blood culture worsens the already existing challenge of isolating an etiologic agent from blood. Clinicians managing the care of pneumonia patients and researchers evaluating the relative contribution of bacterial pathogens to the etiology of these syndromes should make efforts to draw blood culture volumes ≥3 mL, collect specimens prior to antibiotic administration, obtain accurate measurements of antibiotic exposure, and interpret test results in accordance with blood volume and prior antibiotic exposure. Failure to do so may overestimate the proportion of antibiotic-resistant organisms and underestimate the relative contributions of antibiotic-sensitive pathogens, such as *S. pneumoniae*, *H. influenzae*, and *M. catarrhalis*, to pneumonia etiology.

## Supplementary Data

Supplementary materials are available at *Clinical Infectious Diseases* online. Consisting of data provided by the authors to benefit the reader, the posted materials are not copyedited and are the sole responsibility of the authors, so questions or comments should be addressed to the corresponding author. 

## Supplementary Material

DAP_7_15_Supplemental_tables_23Nov16

## Notes

***Author contributions.*** A. J. D. and D. R. M. led the analysis, interpreted results, and drafted the manuscript. M. D. K. and L. L. H. assisted with interpretation of results and drafting of the manuscript. J. A. G. S., H. C. B., W. A. B., D. R. F., L. L. H., S. R. C. H., K. L. K., O. S. L., S. A. M., K. L. O., D. M. T., R. A. K., and D. R. M. conceived and designed the study and supervised study conduct. P. V. A., D. A., A. N. D., B. E. E., C. G., M. M. H., A. Kae., A. Kar., J. M. M., D. P. M., L. M., D. E. P., C. P., J. R., M. S., P. S., S. O. S., and B. T. were involved in study conduct, data collection, and/or data management. S. Z. provided statistical guidance. All authors reviewed and approved the manuscript. A. J. D. and D. R. M. had full access to the data and had final responsibility for the decision to submit for publication.

***Acknowledgments.*** We offer sincere thanks to the patients and families who participated in this study. We acknowledge the significant contributions of the PERCH Study Group and all PERCH investigators. We offer our gratitude to the members of the Pneumonia Methods Working Group, PERCH Expert Group, and PERCH Chest Radiograph Reading Panel for their time and lending expertise to assist the PERCH Study Group (see Supplementary Materials for a full list of names).

***PERCH Study Group.*** Johns Hopkins Bloomberg School of Public Health, Baltimore, Maryland: Katherine L. O’Brien (principal investigator [PI]), Orin S. Levine (former PI, current affiliation: Bill & Melinda Gates Foundation, Seattle, Washington), Maria Deloria Knoll (co-PI), Daniel R. Feikin (joint affiliation with Centers for Disease Control and Prevention [CDC], Atlanta, Georgia), Andrea N. DeLuca, Amanda J. Driscoll, Nicholas Fancourt, Wei Fu, Laura L. Hammitt, Melissa M. Higdon, E. Wangeci Kagucia, Ruth A. Karron, Mengying Li, Daniel E. Park, Christine Prosperi, Zhenke Wu, Scott L. Zeger; Emmes Corporation, Rockville, Maryland: Nora L. Watson; Nuffield Department of Clinical Medicine, University of Oxford, United Kingdom: Jane Crawley; University of Otago, Christchurch, New Zealand: David R. Murdoch; icddr,b, Dhaka and Matlab, Bangladesh: W. Abdullah Brooks (site PI), Hubert P. Endtz, Khalequ Zaman, Doli Goswami, Lokman Hossain, Yasmin Jahan, Hasan Ashraf; Medical Research Council, Basse, The Gambia: Stephen R. C. Howie (site PI), Bernard E. Ebruke, Martin Antonio, Jessica McLellan, Eunice Machuka, Arifin Shamsul, Syed M.A. Zaman, Grant Mackenzie; KEMRI-Wellcome Trust Research Programme, Kilifi, Kenya: J. Anthony G. Scott (site PI and PERCH co-PI), Juliet O. Awori, Susan C. Morpeth, Alice Kamau, Sidi Kazungu, Micah Silaba Ominde; Division of Infectious Disease and Tropical Pediatrics, Department of Pediatrics, Center for Vaccine Development, Institute of Global Health, University of Maryland School of Medicine, Baltimore, Maryland and Centre pour le Développement des Vaccins (CVD-Mali), Bamako, Mali: Karen L. Kotloff (site PI), Milagritos D. Tapia, Samba O. Sow, Mamadou Sylla, Boubou Tamboura, Uma Onwuchekwa, Nana Kourouma, Aliou Toure; Respiratory and Meningeal Pathogens Research Unit, University of the Witwatersrand, Johannesburg, South Africa: Shabir A. Madhi (site PI), David P. Moore, Peter V. Adrian, Vicky L. Baillie, Locadiah Kuwanda, Azwifarwi Mudau, Michelle J. Groome, Nasreen Mahomed; Thailand Ministry of Public Health–US CDC Collaboration, Nonthaburi, Thailand: Henry C. Baggett (site PI), Somsak Thamthitiwat, Susan A. Maloney (former site PI), Charatdao Bunthi, Julia Rhodes, Pongpun Sawatwong, Pasakorn Akarasewi (site co-PI, Ministry of Public Health); Boston University School of Public Health, Boston, Massachusetts and University Teaching Hospital, Lusaka, Zambia: Donald M. Thea (site PI), Lawrence Mwananyanda, James Chipeta, Phil Seidenberg, James Mwansa, Somwe wa Somwe, Geoffrey Kwenda. Canterbury Health Laboratory, Christchurch, New Zealand: Trevor P. Anderson, Joanne Mitchell.

***Financial support.*** PERCH was supported by the Bill & Melinda Gates Foundation (grant number 48968 to the International Vaccine Access Center, Department of International Health, Johns Hopkins Bloomberg School of Public Health). J. A. G. S. was supported by a clinical fellowship from the Wellcome Trust of Great Britain (award number 098532).

***Supplement sponsorship.*** This article appears as part of the supplement “Pneumonia Etiology Research for Child Health (PERCH): Foundational Basis for the Primary Etiology Results,” sponsored by a grant from the Bill & Melinda Gates Foundation to the PERCH study of Johns Hopkins Bloomberg School of Public Health, Baltimore, Maryland.

***Potential conflicts of interest.*** M. D. K. has received funding for consultancies from Merck, Pfizer, and Novartis, and grant funding from Merck. L. L. H. has received grant funding from Pfizer and GlaxoSmithKline. K. L. K. has received grant funding from Merck Sharp & Dohme. S. A. M. has received honoraria for advisory board membership from the Bill & Melinda Gates Foundation, Pfizer, Medimmune, and Novartis; has received institutional grants from GSK, Novartis, Pfizer, Minervax, and the Bill & Melinda Gates Foundation; and has served on speaker’s bureaus for Sanofi Pasteur and GSK. K. L. O. has received grant funding from GSK and Pfizer and participates on technical advisory boards for Merck, Sanofi Pasteur, PATH, Affinivax, and ClearPath. All other authors report no potential conflicts. All authors have submitted the ICMJE Form for Disclosure of Potential Conflicts of Interest. Conflicts that the editors consider relevant to the content of the manuscript have been disclosed.
