# Supplementary material for: The Effect of Antibiotic Exposure and Specimen Volume on the Detection of Bacterial Pathogens in Children With Pneumonia
Source: Clin Infect Dis. 2017 May 27;64(Suppl 3):S368–77. doi: 10.1093/cid/cix101 (PMC5447850; doi:10.1093/cid/cix101)
Supplement: DAP_7_15_Supplemental_tables_23Nov16 [file cix101_suppl_DAP_7_15_Supplemental_tables_23Nov16.pdf]

Supplemental Materials: Antibiotic exposure and blood culture volume

Supplemental Table 1. Proportion of cases and controls with unknown antibiotic exposure status prior to blood specimen collection

	**Cases**		**Controls**	
	**n**	**Unknown (%)**	**n**	**Unknown (%)**
**All Sites**	4,232	167 (4.0)	5,325	461 (8.7)
**Site**				
Kenya	634	49 (7.7)	865	88 (10.2)
Gambia	638	67 (10.5)	654	41 (6.3)
Mali	674	4 (0.6)	725	26 (3.6)
Zambia	617	4 (0.7)	686	66. (9.6)
S. Africa	920	34 (3.7)	966	68 (7.1)
Thailand	224	1 (0.5)	659	94 (14.3)
Bangladesh	525	8 (1.5)	772	59 (7.6)
Age				
<6 months	1,734	107 (6.2)	1,667	180 (10.8)
>6 months	2,498	225 (9.0)	3,658	262 (7.2)
HIV status				
HIV infected	251	6 (2.4)	223	7 (3.1)
HIV uninfected	3,981	326 (8.2)	5,102	435 (8.5)
CXR status				
CXR abnormal	1,935	117 (6.1)	-	-
CXR normal	1,652	173 (10.5)	-	-
Very Severe Pneumonia				
Yes	1,370	123 (9.0)	-	-
No	2,862	228 (7.9)	-	-
Fever				
Yes	3,450	281 (8.1)	-	-
No	782	69 (8.8)	-	-

Supplemental Materials: Antibiotic exposure and blood culture volume

Abbreviation: HIV, human immunodeficiency virus; CXR, chest radiograph

Supplemental Table 2. Association of antibiotic exposure prior to specimen collection with detection of bacterial organisms by culture among children aged 1-59 months enrolled in the PERCH study who had an abnormal chest radiograph and were HIV-uninfected

Specimen	Antibiotic-Exposed[a]	Bacteria isolated: n (%)	Adjusted OR[b] (95%CI)
**Blood (cases)**		**Any bacteria[c]**	
	Yes (n=699)	20 (2.9)	0.42 (0.17, 1.03)
	No (n=936)	35 (3.7)	
		**Any Gram positive bacteria**	
	Yes (n=689)	10 (1.5)	0.47 (0.14, 1.58)
	No (n=919)	18 (2.0)	
		S. pneumoniae	
	Yes (n=682)	3 (0.4)	0.36 (0.08, 1.50)
	No (n=917)	16 (1.7)	
		S. aureus	
	Yes (n=683)	4 (0.6)	0.34 (0.01, 9.92)
	No (n=903)	2 (0.2)	
		**Any Gram negative bacteria**	
	Yes (n=689)	10 (1.4)	0.35 (0.09, 1.34)
	No (n=917)	17 (1.7)	
		H. influenzae	
	Yes (n=681)	2 (0.3))	0.09 (0.01, 1.24)
	No (n=911)	10 (1.1)	
		**Enterobacteriaceae**	
	Yes (n=684)	5 (0.7)	4.48 (0.27, 74.72)

Supplemental Materials: Antibiotic exposure and blood culture volume

Specimen	Antibiotic-Exposed[a]	Bacteria isolated: n (%)	Adjusted OR[b] (95%CI)
	**No (n=903)**	2 (0.2)	
		**Salmonella spp.**	
	**Yes (n=683)**	4 (0.6)	0.67 (0.02, 17.89)
	**No (n=903)**	2 (0.2)	
		**Non fermenting Gram-negative rods**	
	**Yes (n=1,625)**	0 (0.0)	-
	**No (n=2,095)**	0 (0.0)	
**NP (cases)**		***S. pneumoniae***	
	**Yes (n=809)**	335 (41.4)	**0.44 (0.35, 0.56)**
	**No (n=805)**	563 (66.2)	
**NP (controls)**		***S. pneumoniae***	
	**Yes (n=78**	40 (51.3)	**0.47 (0.29, 0.74)**
	**No (n=4,542)**	3,186 (70.2)	
**IS (cases)**		**Any bacteria, excluding normal oropharyngeal flora [d]**	
	**Yes (n=1,243)**	829 (66.7)	**0.22 (0.13, 0.36)**
	**No (n=300)**	279 (93.0)	
		S. pneumoniae	
	**Yes (n=646)**	232 (35.9)	**0.11 (0.06, 0.20)**
	**No (n=208)**	187 (89.9)	
		***H. influenzae* spp.**	**0.16 (0.09, 0.28)**
	**Yes (n=792)**	**378 (47.7)**	

Supplemental Materials: Antibiotic exposure and blood culture volume

Specimen	Antibiotic-Exposed[a]	Bacteria isolated: n (%)	Adjusted OR[b] (95%CI)
	**No (n=230)**	209 (90.9)	
		M. catarrhalis **spp.**	
	**Yes (n=690)**	276 (40.0)	**0.28 (0.15, 0.55)**
	**No (n=152)**	131 (86.2)	
		S. aureus	
	**Yes (n=525)**	111 (21.1)	**0.31 (0.14, 0.66)**
	**No (n=40)**	19 (47.5)	
		**Other Gram-negative rods[e]**	
	**Yes (n=414)**	0 (0.0)	-
	**No (n=21)**	0 (0.0)	

Abbreviation: OR, adjusted odds ratio; NP, nasopharyngeal; OP, oropharyngeal

[a]Antibiotic exposure defined for cases as meeting any of the following criteria: having a positive serum bioassay, received antibiotics at a referral facility, or administered antibiotics at the study facility prior to the collection of specimens (assessed by clinician report and time/date calculation); for controls as having a positive serum bioassay. Participants with unknown antibiotic exposure data are excluded from these analyses.

[b]Relative odds of culture positivity (vs. no growth of any organism) given antibiotic pretreatment, compared to those who are not pretreated. Odds ratios were adjusted for site, severity, blood culture specimen volume >3mL, and age category. Respiratory specimens (NP, IS) in cases were adjusted for the same variables with the exception of blood specimen volume; NP in controls was adjusted for age category and HIV-status.

[c]Excluding contaminants.

[d]No quality stringency criteria applied.

[e]Other gram negative rods isolated from induced sputum includes Acinetobacter spp., *Enterobacter aerogenes*, *Enterobacter cloacae*, Pseudomonas aeruginosa and species, and mixed gram negative rods.

Supplemental Table 3. Association of PCR outcome by antibiotic exposure status among children aged 1-59 months enrolled in the PERCH study who had an abnormal chest radiograph and were HIV-uninfected

Specimen/Test	Antibiotic-Pretreated[a]	PCR Positive n (%)	Adjusted OR[b] (95%CI)	Mean (SD) density (log(10) copies/mL) among positives	Fold Difference[c] (95%CI)
**Whole Blood Pneumococcal PCR - Cases**					
	**Yes (n=655)**	56 (8.5)	1.17 (0.73, 1.91)	2.86 (0.91)	0.20 (-0.16, 0.55)
	**No (n=911)**	64 (7.0)		2.68 (0.77)	
**Whole Blood Pneumococcal PCR - Controls**					
	**Yes (n=81)**	8 (9.9)	1.09 (0.81, 1.48)	2.89 (0.85)	0.43 (-0.19, 1.05)
	**No (n=4,438)**	239 (5.4)		2.34 (0.62)	
**NP/OP Respiratory PCR - Cases**					
		**Any Bacteria**			
	Yes (n= 761)	701 (92.1)	0.69 (0.45, 1.09)	-	-
	No (n= 760)	712 (93.7)			
		S. pneumoniae			
	Yes (n= 760)	526 (69.2)	**0.72 (0.55, 0.93)**	5.37 (1.23)	**-0.49 (-0.66, -0.32)**
	No (n= 759)	574 (75.6)		6.02 (1.26)	
		***S. pneumoniae* above threshold[d]**			
	Yes (n=760)	48 (6.3)	**0.28 (0.19, 0.43)**	7.38 (0.36)	0.10 (-0.03, 0.24)
	No (n=759)	153 (20.2)		7.35 (0.35)	
		H. influenzae			
	Yes (n=759)	397 (52.3)	**0.76 (0.60, 0.98)**	5.61 (1.25)	**-0.35 (-0.54, -0.15)**
	No (n=759)	474 (62.5)		5.96 (1.23)	
		M. catarrhalis			
	Yes (n=759)	484 (63.8)	**0.82 (0.64, 1.07)**	5.14 (1.24)	**-0.39 (-0.56, -0.23)**
	No (n=759)	516 (68.0)		5.64 (1.05)	
		S. aureus			
	Yes (n=760)	133 (17.5)	1.20 (0.86, 1.67)	5.30 (1.12)	0.04 (-0.28, 0.36)
	No (n=759)	105 (13.8)		5.05 (1.05)	
**NP/OP Respiratory PCR - Controls**		**Any Bacteria**			

	Yes (n=61)	56 (91.8)	0.51 (0.20, 1.31)	-	-
	No (n=4,435)	3,793 (96.0)			
		S. pneumoniae			
	Yes (n=61)	38 (62.3)	**0.44 (0.26, 0.76)**	5.26 (1.25)	-0.32 (-0.66, 0.02)
	No (n=3,950)	3,080 (78.0)		5.62 (1.09)	
		***S. pneumoniae* above threshold[d]**			
	Yes (n=61)	1 (1.6)	0.25 (0.03, 1.85)	7.07 (n/a)	-0.19 (-0.79, 0.41)
	No (n=3,950)	286 (7.2)		7.27 (0.30)	
		H. influenzae			
	Yes (n=61)	31 (50.8)	1.22 (0.72, 2.05)	5.13 (1.06)	-0.32 (-0.69, 0.36)
	No (n=3,947)	2,076 (52.6)		5.59 (1.05)	
		M. catarrhalis			
	Yes (n=61)	43 (70.5)	**0.75 (0.42, 1.34)**	5.07 (1.34)	**-0.21 (-0.60, -0.01)**
	No (n=3,947)	2,938 (74.4)		5.50 (1.00)	
		S. aureus			
	Yes (n=61)	8 (13.1)	1.00 (0.46, 2.19)	5.17 (1.31)	0.06 (-0.66, 0.78)
	No (n=3,950)	543 (13.8)		4.97 (1.05)	
**Induced Sputum PCR - Cases**		**Any bacteria**			
	Yes (n=1,136)	1,005 (88.5)	**0.43 (0.19, 0.97)**	-	-
	No (n=258)	248 (96.1)			
		S. pneumoniae			
	Yes (n=1,133)	755 (66.6)	0.68 (0.45 1.04)	4.98 (1.13)	**-0.26 (-0.44, -0.08)**
	No (n=258)	203 (78.7)		6.25 (1.09)	
		H. influenzae			
	Yes (n=1,133)	560 (49.4)	**0.61 (0.42, 0.91)**	5.17 (1.14)	**-0.28 (-0.48, -0.09)**
	No (n=258)	190 (73.6)		6.13 (1.12)	
		M. catarrhalis			
	Yes (n=1,133)	609 (53.7)	**0.61 (0.42 0.91)**	4.78 (1.28)	**-0.21 (-0.42, -0.01)**
	No (n=258)	190 (73.6)		5.97 (1.22)	
		S. aureus			
	Yes (n=1,133)	250 (17.7)	0.82 (0.48, 1.41)	4.97 (1.11)	-0.06 (-0.44, 0.32)
	No (n=258)	273 (14.1)		5.10 (0.86)	

Supplemental Materials: Antibiotic exposure and blood culture volume

Abbreviations: NP/OP, nasopharyngeal/oropharyngeal; PCR, polymerase chain reaction; OR, odds ratio

[a] Antibiotic exposure defined for cases as any of the following: having a positive serum bioassay, received antibiotics at a referral facility, or received antibiotics at the study facility prior to the collection of specimens (assessed by clinician report and time/date calculation); Antibiotic exposure for controls is defined as having a positive serum bioassay. Participants with unknown antibiotic exposure status are excluded from analyses.

[b]Relative odds of laboratory outcome given antibiotic exposure, compared to those who were not pretreated. Odds ratios are adjusted for site, severity, CXR status, HIV-infection status, and age. NP/OP outcomes in controls are adjusted for site, HIV-infection status, and age.

[c]Fold-difference in mean log10 transformed PCR density (copies/mL) for pretreated compared to non-pretreated individuals, among those PCR positive.

[d]Above PCR density threshold (6.9 log10 copies/mL) associated with case status (Baggett et al., this supplement).

Acknowledgements:

PERCH Expert Group. William C. Blackwelder, Harry Campbell, John A. Crump, Adegoke Falade, Menno D. de Jong, Claudio Lanata, Kim Mulholland, Shamim Qazi, Cynthia G. Whitney.

Pneumonia Methods Working Group. Robert E Black, Zulfiqar A Bhutta, Harry Campbell, Thomas Cherian, Derrick W Crook, Menno D de Jong, Scott F Dowell, Stephen M Graham, Keith P Klugman, Claudio F Lanata, Shabir A Madhi, Paul Martin, James P Nataro, Franco M Piazza, Shamim A Qazi, and Heather J Zar.

PERCH Chest Radiograph Reading Panel
Readers: Dr. Kamrun Nahar, Dr. Fariha Bushra Matin, Dr. Claire Oluwalana, Dr. Bernard Ebruke, Dr. Joyce Sande, Dr. Micah Silaba Ominde, Dr. Mahamadou Diallo, Dr. Breanna Barger-Kamate, Dr. Nasreen Mahomed, Dr. David P. Moore, Dr. Anchalee Kruatrachue, Dr. Piyarat Suntarattiwong, Dr. Musaku Mwenechanya, Dr. Rasa Izadnegahdar, **Arbitrators:** Dr. Vera Manduku, Dr. John DeCampo, Dr. Marg DeCampo, Dr. Fergus Gleeson.

PERCH Contributors:
Bangladesh: Kamrun Nahar, Arif Uddin Sikdir, Sharifa Yeasmin, Dilruba Ahmed, Muhammad Ziaur Rahman, Muhammad Yunus, Muhammad Al Fazl Khan, Muhammad Jubayer Chisti, Abu Sadat Muhammad Sayeem, Shahriar Bin Elahi, Mustafizur Rahman; **The Gambia:** Michel Dione, Emmanuel Olutunde, Peter Githua, Ogochukwu Ofordile, Rasheed Salaudeen, David Parker; **Kenya:** Shebe Mohamed, Siti Ndaa, Micah Silaba, Neema Muturi, Angela Karani, Sammy Nyongesa, Anne Bett, Daisy Mugo, Salim Mwarumba, Robert Musyimi, Andrew Brent, James Nokes, David Mulewa, Joyce Sande, John Odhiambo, Joshua Wambua, Nuru Kibirige, Caroline Mulunda, Hellen Mjalla, Norbert Katira, Karen Dama, Loice Masha, Christine Mutunga, Mwanajuma Ngama, Stephen Mangi, Riziki Anthony, Mwarua Yubu, Elijah Wakili, Benson Katana, Shoboi Mgunya, Emmanuel Mumba, Benedict Mver, George Kuria, Felix Githinji, Norbert Kihuha, Boniface Jibendi, Tahreni Bwanaali, Agustus Kea; **Mali:** Nana Kourouma, Aliou Toure, Mahamadou Diallo, Breana Barger-Kamate, Mariam Samake, Seydou Sissoko, Abdoul Aziz Maiga, Mariam Samake, Toumani Sidibe, Mariam Sylla, Aziz Diakite, Bassirou Diarra; **South Africa:** Azwidihwi Takalani, Andrea Hugo, Susan Nzenze, Ndulela Titi, Mmabatho Selela, Malebo Motiane, Minah Nkuna,Nonhlanhla Tsholetsane, Sibonsile Moya, Debra Katisi, Tondani Netshishivhe, Lerato Mapetla, Gudani Singo, Simphiwe Gasa, Cece Mgenge, Nozipho Mthunzi, Nombulelo Monedi, Tanja Adams, Shafeeka Mangera, Jeannette Wadula, Peter Tsaagane, Jenifer L. Vaughan, Sakina Loonat, Martin Hale, Sugeshnee Pather, Mariëtte Middel, Siobhan Trenor, Palesa Morailane, Ntombi Maya, Rene Sterley, Charné Combrinck, Given Malete, Lerato Qoza, Grizelda Liebenberg, Hendrik van Jaarsveld, Zunaid Kraft, Lisa-Marie Mollentze, Lourens Combrinck, Tsholofelo Mosome; **Thailand:** Sununta Henchaichon, Dr. Tussanee Amornintapichet, Dr. Somchai Chuananont, Toni Whistler, Juraiporn Ratanodom, Patranuch Sapchookul, Ornuma Sangwichian, Sirirat Makprasert, Manoon Hirunsalee, Possawat Jorakate, Anek Kaewpan, Duangkamol Siludjai, Apiwat Lapamnouysup, Dr. Wantana Paveenkittiporn, Waraporn Ubonphen, Dr. Peera Areerat, Ms.Yupapan Wannachaiwong, Ms Tewa Faipet, Ms Punnat Natnarakorn, Ms Ahchanan Sacharone, Mr.Winai Makmool, Ms. Kanlaya Sornwong, Ms. Promporn Sansuriwong, Ms. Ratchanida Potiya, Ms. Wasana Hongsawong, Ms.Wipa Matchaikhen, Ms. Thatsanawan Chaiyabil, Ms.Piyapai Wannarach, Ms Chamaiporn Wadeesirisak, Mr. Yuttapong Norapet, Mattana Bangkung, Mr. Barameht Piralam, Sathapana Naorat, Anchalee Jatapai, Prasong Srisaengchai, Dr. Leonard Peruski, Ms.Dawan

Phaensoongnoen, Ms.Tussaaorn Klangprapan, Ms.Narawadee Dumrongdee, Ms.Atchara Srithongkham, Mr. Piyawut Noinont, Ms. Pornthip Kamlee, Ms.Siyapa Mongkornsuk; **Zambia:** Justin Mulindwa, Musaku Mwenechanya, John Mwaba, Magdalene Mwale, Julie Duncan, Kazungu Siazele, Muntanga Mapeni, Emily Hammond; **Canterbury Health Laboratory, Christchurch, New Zealand:** Rose Watt, Shalika Jayawardena.
